# Concentration Gradient Constructions Using Inertial Microfluidics for Studying Tumor Cell–Drug Interactions

**DOI:** 10.3390/mi11050493

**Published:** 2020-05-12

**Authors:** Shaofei Shen, Fangjuan Zhang, Mengqi Gao, Yanbing Niu

**Affiliations:** College of Life Science, Shanxi Agricultural University, Taigu 030801, China; panglong2012@nwsuaf.edu.cn (F.Z.); tianchang984@nwsuaf.edu.cn (M.G.)

**Keywords:** microfluidic chip, inertial microfluidics, drug screening, concentration gradient, spiral mixer

## Abstract

With the continuous development of cancer therapy, conventional animal models have exposed a series of shortcomings such as ethical issues, being time consuming and having an expensive cost. As an alternative method, microfluidic devices have shown advantages in drug screening, which can effectively shorten experimental time, reduce costs, improve efficiency, and achieve a large-scale, high-throughput and accurate analysis. However, most of these microfluidic technologies are established for narrow-range drug-concentration screening based on sensitive but limited flow rates. More simple, easy-to operate and wide-ranging concentration-gradient constructions for studying tumor cell–drug interactions in real-time have remained largely out of reach. Here, we proposed a simple and compact device that can quickly construct efficient and reliable drug-concentration gradients with a wide range of flow rates. The dynamic study of concentration-gradient formation based on successive spiral mixer regulations was investigated systematically and quantitatively. Accurate, stable, and controllable dual drug-concentration gradients were produced to evaluate simultaneously the efficacy of the anticancer drug against two tumor cell lines (human breast adenocarcinoma cells and human cervical carcinoma cells). Results showed that paclitaxel had dose-dependent effects on the two tumor cell lines under the same conditions, respectively. We expect this device to contribute to the development of microfluidic chips as a portable and economical product in terms of the potential of concentration gradient-related biochemical research.

## 1. Introduction

Globally, cancer is widespread and affects one-third of the world’s population [1,2]. It is a major public health problem worldwide [3]. In the past few decades, a variety of in vitro tumor models have been developed for drug testing and screening, but there is no simple and effective system for timely screening of anticancer drugs and dose-response evaluation of tumor patients [4,5]. The routine drug development process for checking drug specificity and toxicity mainly involves drug screening in preclinical trials, in vitro platforms, and animal models [6,7,8]. However, these methods have certain limitations and cannot effectively mimic the microenvironment of tumor cell growth [9]. To significantly reduce the failure rate of clinical tumor trials and related costs of in vitro model preparation, it is necessary to develop a tumor model that is more time-saving, efficient, and low-cost.

The progress of microfluidic technology provides a new way for constructing tumor models due to its good biocompatibility and light transmittance [10]. Prepolymers for fabricating microfluidic chips are readily available and inexpensive. Also, due to the design advantage of microfluidic devices at the micron level, the cell microenvironment can be effectively simulated [11,12]. Microfluidic technology has been effectively applied for the establishment of concentration gradient, simulation of the tumor cell metastasis process, and screening of the anti-tumor drug [13]. In particular, the construction of a “tumor on a chip” based on drug-concentration gradient generator has greatly expanded worldwide and has been widely accepted by pharmaceutical companies as a tool for drug development [14]. These technologies could rapidly form a drug gradient for a long time by geometric design and precisely monitor the cell physiological process in real-time [15].

The drug-concentration gradient generators mainly include flow-based and diffusion-based gradient generator [16,17]. Therefore, the flow-based gradient generator on the strength of “Christmas tree structure” has attracted wide attention [18,19,20]. However, the linear drug-concentration gradients and narrow concentration ranges generated by this gradient generator severely limited the application of microfluidics in drug screening. Hence, Zhang et al. presented a “Christmas tree mixer” structure with non-uniform channel sizes to achieve a logarithmic mixing ratio gradient [21]. It also has a wider concentration range. However, this structure is more complicated in its pipeline design. Additionally, some groups use complex valve operation and interface system to solve these problems, but these devices are not appropriate for the conventional application of drug screening [22,23]. Furthermore, these devices require specific and sensitive flow rates to achieve efficient mixing, which increases the difficulty of drug screening and the inaccuracy of the quantitative concentration gradients [24,25,26]. Microfluidic-based inertial systems have been increasingly studied for fluid/cell manipulations [27,28,29]. Our group has recently proposed a unique inertial microfluidics method for adjusting Dean vortexes to achieve highly efficient fluid manipulation and cell separation [26,30,31]. A three-set gradient generator based on Dean flow is developed to increase the mixing area and decrease the mixing lengths [32]. This device is a new type of flow-independent microfluidic chip that can be used to quickly build stable, predictable, and controllable gradients with low background concentrations. However, the device is mainly used to evaluate the individual interactions and combinatory interactions of different anticancer drugs on one tumor cell line. The range of concentration-gradient formation in the device is also narrow, being similar to previous gradient generator studies (4 or 5 concentration-gradient formations) [16,33,34]. Also, a microfluidic device that achieves more simple and easy to operate concentration gradient constructions with insensitive flow rate for studying the drug’s effect on two tumor cell lines remains largely out of reach.

Here, we developed an easy and compact flow-rate insensitive microfluidic system, which can quickly construct an efficient and reliable drug-concentration gradient with a wide range of flow rates for studying tumor cell–drug interactions. The single-layer device needs only a simple construction and it also reduces operational complexity and environmental interference in cell culture. The study of the dynamics of concentration-gradient formation based on a unique double spiral mixer was investigated systematically and quantitatively. Accurate, stable, and controllable dual drug-concentration gradients were produced to evaluate simultaneously the efficacy of the anticancer drug against two tumor cell lines (human breast adenocarcinoma cells and human cervical carcinoma cells). The changes in cellular activity under different doses of drug were observed and analyzed in real time. Our device has the distinct advantage of being able to simultaneously assess the individual effects of a drug on both tumor cell lines for a wide range of drug-concentration screening based on a limited rather than sensitive flow condition, which is beneficial to quantitatively and accurately evaluate the best drug concentration at a large scale and has the potential to promote the development of drug screening of innovative anti-cancer therapeutics. 

## 2. Materials and Methods

### 2.1. Materials and Reagents

RTV 615 poly (dimethylsiloxane) (PDMS) pre-polymer and curing agent were purchased from Momentive Performance Materials(Waterford, NY, USA); Surface-oxidized silicon wafers were from Shanghai Xiangjing Electronic Technology, Ltd. (Shanghai, China); AZ 50XT photoresist and developer were from AZ Electronic Materials (Somerville, NJ, USA); paclitaxel, fluorescein diacetate (FDA), penicillin-streptomycin stabilized solution were from Sigma-Aldrich (St. Louis, MO, USA); acridine orange (AO) and propidium iodide (PI) was purchased from Keygen Biotech (Nanjing, China); Dulbecco’s modified Eagle’s medium (DMEM), fetal bovine serum (FBS), trypsin and phosphate-buffered saline (PBS) were from Gibco Invitrogen Corporation (Gran Island, NY, USA). Fibronectin (FN) was from Solarbio (Beijing, China). The analytical reagent-grade solvents and other chemicals were bought from local commercial suppliers, unless otherwise stated. All solutions were prepared using ultra-purified water supplied by a Milli-Q system (Millipore^®^, Burlington, MA, USA). Human breast adenocarcinoma cells (MCF-7) and human cervical carcinoma cells (HepG2) were kindly supplied by Stem Cell Bank, Chinese Academy of Sciences.

### 2.2. Concentration Gradient Stability of the Microfluidic Devices

In order to determine the stability of the microfluidic system and the cell culture cycle in vitro, we studied the concentration gradient formed by the microfluidic device. Firstly, FDA and PBS were continuously perfused from inlet 1 and inlet 2 by injection pump. After that, the fluorescence intensity of each microchamber was monitored by photographic equipment every 12 h. Finally, image-pro1 Plus 6.0 (Media Cyternetics, Silver Spring, MD, USA) and origin 9 (origin Inc.) were used to investigate and analyze the formation of concentration gradient in each microchamber.

### 2.3. The Coefficient of Variation

The coefficient of variation (CoV) = *σ_c_*/*μ* is defined as the ratio of standard deviation of normalized concentration (*σ_c_*) to the mean concentration (*μ*). *σ_c_* and *μ* were calculated using the following equations:

(1) $$\sigma_{c}=\sqrt{\frac{\sum_{i}^{n}{{(C}_{i}-\mu )}^{2}}{n}}$$

(2) $$\mu =\frac{1}{n}\sum_{i}^{n}C_{i}$$

where *C_i_* denotes the concentration at each datum point (*i*) and *n* is the total number of data points. The CoV is used to measure the dispersion of species within the region of interest. For complete mixing, CoV = 0, and for no mixing, CoV = 1. Experimental CoV values were compared with the simulation results. Furthermore, we utilized a CoV value of 0.1 as the upper-level threshold for acceptable mixing.

### 2.4. Experimental Setup

The dye/medicine/cell sample is introduced into the microfluidic device via a syringe pump. It forms a continuous and stable flow state. Connect two 5 mL syringes to the two inlets (diameter: 2 mm) of the device using a Tygon tube (inner diameter: 0.42 mm; length: 30 cm). After irradiating with ultraviolet (UV) light for 2 h, the device was rinsed with 75% ethanol for 2–3 min; finally, it was rinsed with PBS working buffer. Both inlets were tested at the same flow rate. We used specific equipment in different batches. Each experiment was repeated at least 10 times.

### 2.5. Microscopy and Image Analysis

Cell monitoring and fluorescence observations were performed using an inverted microscope (Olympus, CKX41, Tokyo, Japan), a camera with a charge-coupled device (Olympus, DP72), and a mercury lamp (Olympus, URFLT50). Image-pro1 Plus 6.0 (Media Cyternetics, Silver Spring, MD) and origin 9 (origin Inc.) were used for image and data analysis.

### 2.6. Cell Perfusion

The microfluidic device was sterilized under UV conditions for 2 h, and then the microchamber used for cell culture was treated with 50 μg·mL^−1^ FN solution and treated at 37 °C for 4 h. The excess FN solution was washed with PBS buffer. MCF-7 and HepG2 cell suspensions with a cell density of 1.0 × 10^7^ cells mL^−1^ were pumped into the microchamber from the corresponding outlet at a flow rate of 0.2 μL·min^−1^. MCF-7 cells and HepG2 cells were inoculated in 18 different chambers. After generating the uniform distribution of cells, the perfusion was no longer continuous. When the cells grew adherent with a similar number of total cells (≈1.0 × 10^5^ cells) in each microchamber, DMEM containing paclitaxel (1 mg·mL^−^^1^) and drug-free DMEM were injected into inlet 1 and inlet 2. The medium reached the microchamber by the spiral mixer, and the cells in the microchamber were treated with different concentration gradients. The effect of the drug on tumor cells was observed 24 h after the drug was administered. Then, the drug-containing medium was removed and washed with PBS 2–3 times. AO/PI staining solution was introduced into the microchamber at a flow rate of 10 μL·min^−1^, stained at room temperature for 10 min, and then washed with PBS 3–5 times. Finally, fluorescence images were taken in each microchamber to assess the effects of different doses of paclitaxel on the viability of MCF-7 cells and HepG2 cells. Cell viability is defined as follows:

Cell viability = (Live cells/Total cells) × 100%(3)

## 3. Results and Discussion

### 3.1. Device Design

**Figure 1:**
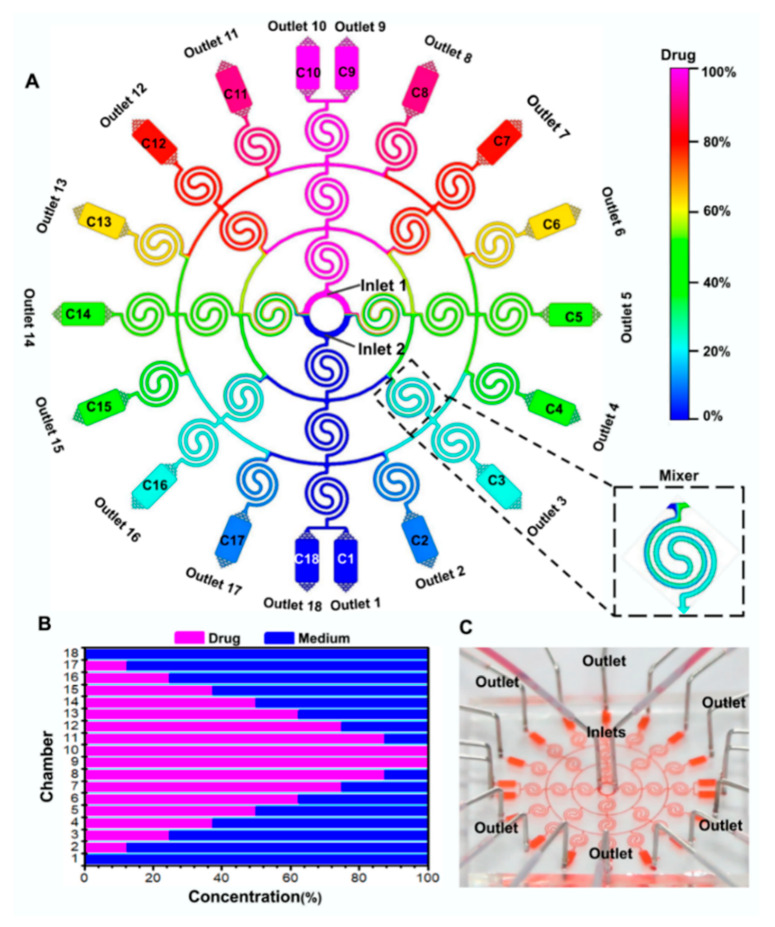
Schematic diagram of concentration gradient construction in the designed device under a flow rate of 20 μL·min^−1^. (**A**) Simulation images of different concentration gradients in the device. (**B**) Quantitative characterization of the concentration gradient formed in the microchamber of each terminal of the device. (**C**) Actual microfluidic device. The food dye solution (red) is loaded in the designed microfluidic device from the inlets.

In this study, we designed a microfluidic device containing 18 microchambers (1200 μm width; 2200 μm length; 50 μm height) and 28 spiral mixers (150 μm width; 50 μm height) formed by double spiral channels (see Figure 1A,C). The study of cell culture was carried out in the microchamber. The inlets and outlets of the microfluidic device were used for injection and elimination procedures. 16, 8 and 4 spiral mixers were connected in three circles (150 μm width; 50 μm height) respectively. The micromixers were able to completely mix the drug and medium, resulting in the successful formation of two sets of the same drug-concentration gradients in the microchambers (see Figure 1A). In addition, a horizontal bar chart (see Figure 1B) was drawn to show the simulation percentages of drug-concentration gradients (namely, chamber 1: negative control; chamber 2: 12.5% drug; chamber 3: 25% drug; chamber 4: 37.5 % drug; chamber 5: 50% drug; chamber 6: 62.5% drug; chamber 7: 75% drug; chamber 8: 87.5% drug; chamber 9: 100% drug; chamber 10: 100% drug; chamber 11: 87.5% drug; chamber 12: 75% drug; chamber 13: 62.5% drug; chamber 14: 50% drug; chamber 15: 37.5% drug; chamber 16: 25% drug; chamber 17: 12.5% drug; chamber 18: negative control). The establishment of drug-concentration gradients in the device provides a basis for subsequent cell culture-based drug assays.

### 3.2. Dual Concentration-Gradients Formation with a Wide Range of Flow Rates

**Figure 2:**
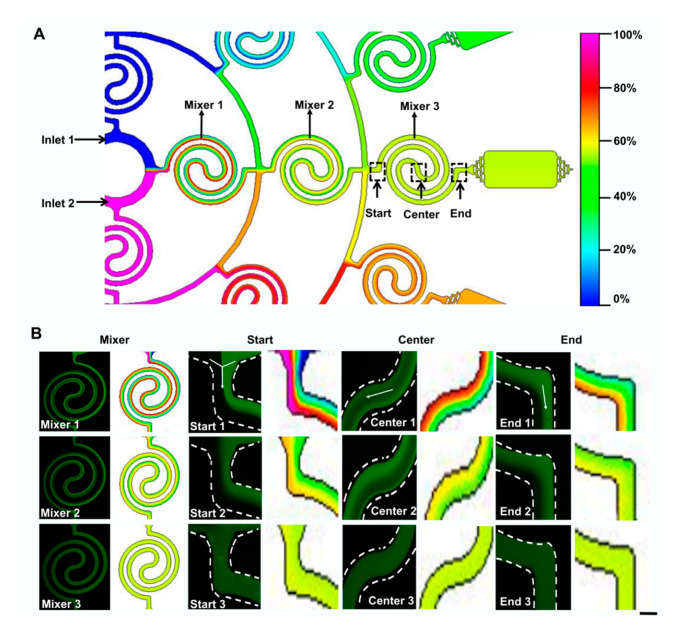
Mixing effect of the designed microfluidic device with successive spiral mixer regulations at a flow rate of 20 μL·min^−1^. (**A**) Simulation imaging of the microfluidic device. Three areas (start, center, and end) in three spiral mixers were used to identify the mixing status. (**B**) Fluorescence and simulation images of different regions of three spiral mixers. Every two adjacent white dotted lines delineate the boundary of the microchip. Scale bar, 150 μm.

To demonstrate the ability of our designed device for manipulating dual concentration-gradient formation, the dynamic process of the mixing experiment by spiral mixers in the device is simulated by a computer under a certain flow rate. As shown in Figure 2A, two different source solutions were added to inlet 1 and inlet 2 to mix through the spiral mixers. The simulation results showed that after passing through three spiral mixers, the two source solutions could achieve a good mixing effect. Although the color was uneven in the Mixer 1 and Mixer 2, good mixing in Mixer 3 was achieved. The successive spiral mixers could help to improve the mixing effect by increasing mixing time (see Figure 2B). To confirm the conclusion, fluorescein hybrid experiments, introducing FDA from inlet 1 and PBS from inlet 2, were carried out under the same flow rate (see Figure 2B). The results also demonstrated that the mixing states became better with variable-length. In general, the ideal form of mixing state by successive spiral mixer regulations could be realized.

**Figure 3:**
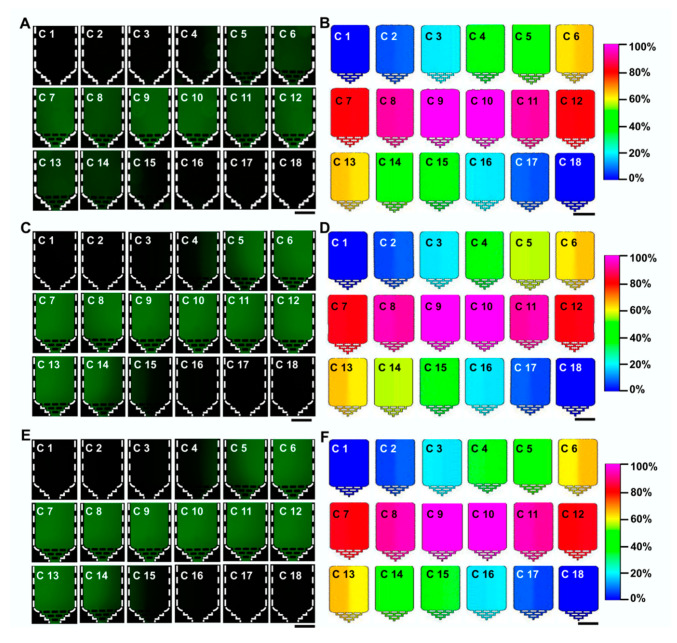
Formation of concentration gradients in 18 microchambers under different flow conditions. (**A**,**C**,**E**) The mixed states of fluorescein dye at different flow rates of 10 μL·min^−1^ (**A**), 20 μL·min^−1^ (**C**), and 50 μL·min^−1^ (**E**). Every two adjacent white dotted lines delineate the boundary of the microchip. (**B**,**D**,**F**) Computer simulation effect at the flow rates of 10 μL·min^−1^ (**B**), 20 μL·min^−1^ (**D**), and 50 μL·min^−1^ (**F**). Scale bar, 500 μm.

To further assess the effect of operating conditions on the formation of dual drug-concentration gradients, we conducted fluorescein mixing experiments and computer simulation at different flow rates to explore the distribution of concentration gradients in 18 microchambers (see Figure 3). The results showed that the mixing effect in each microchamber was good on the whole under the flow rates of 10 μL·min^−1^, 20 μL·min^−1^, and 50 μL·min^−1^. The successive spiral mixer regulations in our designed device can achieve a decent and stable mixing state. More importantly, precise concentration gradients were stably fabricated in each terminal microchamber. Two groups of the same concentration gradients can be observed (namely, chamber 1: chamber 18; chamber 2: chamber 17; chamber 3: chamber 16; chamber 4: chamber 15; chamber 5: chamber 14; chamber 6: chamber 13; chamber 7: chamber 12; chamber 8: chamber 11; chamber 9: chamber 10), which demonstrate that the device could produce an excellent mixing performance that is not influenced by a wide range of flow rates. In particular, our previous study demonstrated the flow rate range (≤50 μL·min^−1^) in the compact double spiral mixer could be considered to avoid high shear stress that damages tumor cell adhesion and proliferation [32]. So the maximum shear stresses in the 18 microchambers at the different flow rates of 10 μL·min^−1^, 20 μL·min^−1^, and 50 μL·min^−1^ cannot affect culture-based cell activity when continuously used.

**Figure 4:**
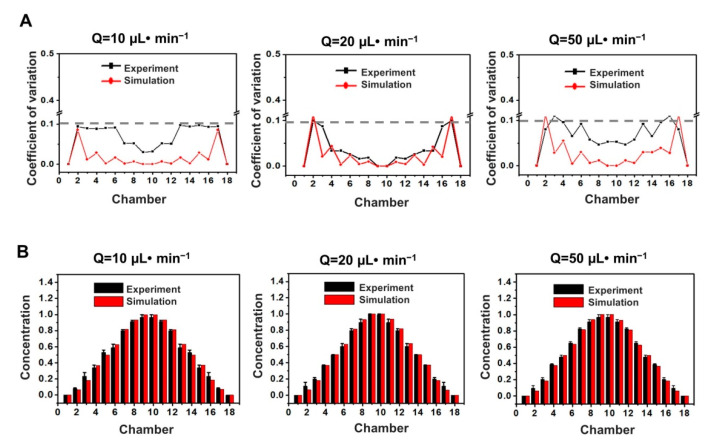
Quantitative gradients characterization in each microchamber under different flow rates of 10 μL·min^−1^, 20 μL·min^−1^, and 50 μL·min^−1^. (**A**) Experimental and computer-simulated coefficient of variation (CoV) values in 18 microchambers. (**B**) Experimental and computer-simulated concentration values in 18 microchambers. Standard deviations deduced from 10 parallel experiments are shown as the error bars. Scale bar, 500 μm.

Furthermore, to demonstrate the capability of our designed device to manipulate concentration-gradient formation, a coefficient of variation (CoV) of value 0.1 was usually used as the upper limit of the acceptable mixing value [26]. Good mixing can be observed under different flow rates of 10 μL·min^−1^, 20 μL·min^−1^, and 50 μL·min^−1^ (see Figure 4A). To be specific, the CoV value in the resulting in the successful formation of two sets of the same drug-concentration gradients in the microchamber was close to 0.1 at the flow rate of 10 μL·min−1. If the flow rate was increased to 20 μL·min^−1^, the CoV value in the resulting in the successful formation of two sets of the same drug-concentration gradients in the microchamber was the smallest. When at a higher flow rate of 50 μL·min^−1^, the CoV value in the resulting in the successful formation of two sets of the same drug-concentration gradients in the microchamber increased again. This phenomenon is possible because sufficient mixing cannot be accomplished with small dean vortices or short mixing time. These demonstrated that lower or higher flow rates were not conducive to the formation of the best mixing state. In addition, the experimental and simulation value of concentration-gradients in each resulting in the successful formation of two sets of the same drug-concentration gradients in the microchamber was quantitatively analyzed under different flow rate conditions (see Figure 4B). When the flow rate was 10 μL·min^−1^, 20 μL·min^−1^, and 50 μL·min^−1^, the 18 microchambers in the microfluidic device formed two sets of symmetrical concentration gradients. The experimental values were consistent with computer simulations. These results indicated that the effective dual drug-concentration gradients could be constructed in a flow-rate insensitive microfluidic system.

**Figure 5:**
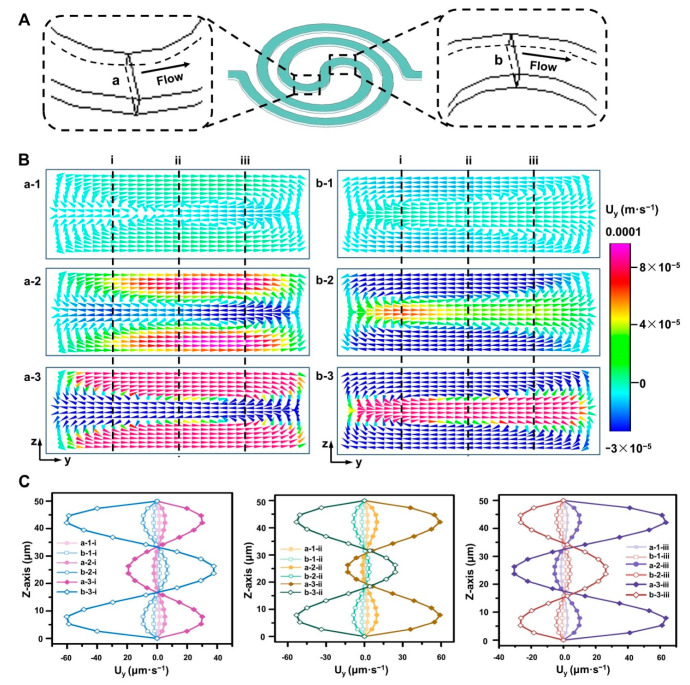
Dean flow induced by the double spiral mixer. (**A**) Schematic of the spiral mixer channel. (**B**) Computer simulation of Dean flow formations under different flow conditions. a and b respectively represent the fluid velocity field of the channel cross-section corresponding to the positions in Figure 5A. The flow rates are 10 μL·min^−1^, 20 μL·min^−1^, and 50 μL·min^−1^ (corresponding to the channel cross-sectional flow rates of a-1 and b-1, a-2 and b-2, and a-3 and b-3 in Figure 5B, respectively). (**C**) Quantitative analysis of Dean flow in the cross-section. The results were obtained at the positions shown by the dotted lines in Figure 5B.

To further clarify the causes and confirm the reliability of the above results, the Dean flow simulation of two cross-sections (see Figure 5A) in our unique spiral mixer is carried out. As shown in Figure 5B, varying Dean flow distributions in the top and bottom halves of the spiral mixer channel were generated under different flow rates. To better distinguish the dynamics of flows in two sections quantitatively, we quartered the cross-section of the concerned region longitudinally (z-direction) aiming to collect the calculated point velocity along the border (i, ii, and iii in Figure 5B) of each of the two neighboring parts. As shown in Figure 5B and 5C, a Dean flow with higher fluid velocity field in the y-axis (*U*_y_) is gradually formed with the growth of flow rate. As we know, Dean number (*De*) can represent the magnitude and qualitative characteristic of Dean flow. *De* is positively correlated to *U*_y_ [30,32]. It indicates that the strength of Dean flow will increase under high flow-rate conditions. The resulting Dean flow acceleration can improve the diffusion transport and effectively enhance the mixing effect in a short time. It is worth noting that the overall mixing effect will become weaker when the flow rate (50 μL·min^−1^) is too high (see Figure 4A), indicating that the acceleration of Dean flow cannot completely eliminate the negative effect of short reaction time on the mixing effect. Therefore, we should have certain requirements on the flow rate in the actual operation process. On the whole, the results show that our microfluidic device has good mixing performance in a wide range of flow rates. It is also able to precisely construct the required concentration gradient, which will lay the foundation for further studies on the responses of tumor cells to dual drug-concentration gradients based on the device.

### 3.3. Evaluation of Drugs on Cell Viability

**Figure 6:**
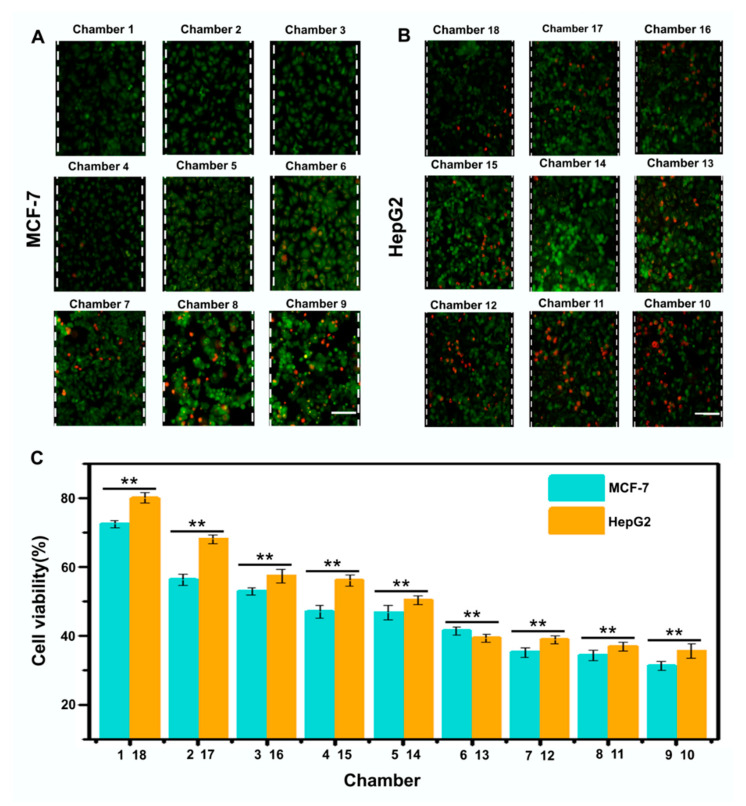
Responses of two tumor cells (human breast adenocarcinoma cells (MCF-7) and human cervical carcinoma cells (HepG2)) to different drug-concentration gradients (paclitaxel). (**A**,**B**) Acridine orange/propidium iodide (AO/PI)-stained fluorescence images of MCF-7 cells (A) and HepG2 cells (**B**) in different microchambers after continuous drug action of different concentrations for 24 h. (**C**) Comparison of cell viability of MCF-7 cells and HepG2 cells. Standard deviations deduced from ten parallel experiments are shown as the error bars, with the significance assessed by analysis of variance (ANOVA).*P < 0.05 and **P < 0.01. Scale bar, 400 μm.

Paclitaxel is an antitumor drug, which can stimulate the phosphorylation of β-tubulin in differentiated and undifferentiated cells, increase microtubule polymerization, and lead to cell death [35,36,37,38]. The drug-containing medium containing paclitaxel (1 mg·mL^−1^) was introduced from inlet 1, and the ordinary medium was introduced from inlet 2. The successive spiral mixers in the microfluidic device were used for mixing to form a series of stable and controllable drug-concentration gradients (namely, chamber 1: 0 mg·mL^−1^; chamber 2: 0.125 mg·mL^−1^; chamber 3: 0.25 mg·mL^−1^; chamber 4: 0.375 mg·mL^−1^; chamber 5: 0.5 mg·mL^−1^; chamber 6: 0.625 mg·mL^−1^; chamber 7: 0.75 mg·mL^−1^; chamber 8: 0.875 mg·mL^−1^; chamber 9: 1 mg·mL^−1^; chamber 10: 1 mg·mL^−1^; chamber 11: 0.875 mg·mL^−1^; chamber 12: 0.75 mg·mL^−1^; chamber 13: 0.625 mg·mL^−1^; chamber 14: 0.5 mg·mL^−1^; chamber 15: 0.375 mg·mL^−1^; chamber 16: 0.25 mg·mL^−1^; chamber 17: 0.125 mg·mL^−1^; chamber 18: 0 mg·mL^−1^). To evaluate the effects of different doses of paclitaxel on MCF-7 and HepG2 tumor cells, the cells in the two microchambers (1,18) as a control group were treated with the normal medium for 24 h. For cells treated with the drug-containing medium, cell growth was inhibited. Compared with the cells which grew normally adherently in the control microchambers, cells culturing in other microchambers showed varying degrees of apoptosis and necrosis (see Figure 6A). Additionally, cell viability decreased significantly with the increase of drug concentration and dose, which demonstrated that drug-concentration gradients could influence the growth activity of tumor cells (see Figure 6A,B). It also showed that paclitaxel inhibited MCF-7 cells more strongly than HepG2 cells (see Figure 6B), because of the stronger cytotoxic influence of drug-concentration gradients on MCF-7 cells [39,40]. In fact, drug treatment has a negative correlation with cell viability that corresponds to the formative drug-concentration gradients. The paclitaxel had dose-dependent effects on the two tumor cell lines under the same conditions, respectively. Therefore, the above research shows that the device can effectively perform cell culture and the formation of concentration gradients, and realize the simultaneous evaluation of the effect of one drug on two tumor cell lines. 

To verify the reliability of our equipment in drug screening, we used the concentration values obtained from the simulated concentration to perform traditional cell cultures in Petri dishes. As shown in Figure S1, the survival rates of MCF7 cells and HepG2 cells in microfluidics under different paclitaxel concentration gradients were linearly related to those of conventional methods. At the same drug concentration, the survival rates of the two tumor cell lines were the same as that in the Petri dish, indicating that the platform could replace the traditional cell culture with a good potential for drug screening. Although there are commercially available culture methods that can be used to culture cells in more than 18 culture chambers, traditional devices frequently require a lengthy operating time and cannot enable tumor cells to obtain continuous nutrient supply and drug treatments, and remove cell wastes to actualize a dynamic microenvironment more realistically and effectively in the long term. Our device can solve the above problems and achieve broader range drug-concentration screening and cell culture analysis by designing more circles and mixers [41]. Our equipment is simple and convenient with high precision and low operating requirements for staff. Therefore, it has great development potential in cell culture and drug screening, and solves problems that occur during the operation of traditional equipment.

## 4. Conclusions

In this study, we demonstrated a microfluidic device based on successive spiral mixer regulations that can be used for cell culture and drug screening. The operation conditions were studied under different flow rates. CoV and Dean flow were used to describe the concentration gradient kinetics and mixing effect quantitatively. The results showed that the device had good mixing performance over a wide flow range (10 μL·min^−1^, 20 μL·min^−1^, and 50 μL·min^−1^). It could produce two stable and effective drug-concentration gradients through successive spiral mixers. At the same time, the tumor cell (MCF-7 and HepG2 cells) survival rate in the 18 microchambers decreased with the increase of drug-concentration gradients. This result proves that our device can realize drug screening of dual concentration-gradients under the condition of wide flow rates and easy operation, which can effectively reduce a series of tedious operations in cell-culture and drug screening. The preparation and operation of this microfluidic device require no higher requirements. Lower concentration gradient constructions and broader range drug-concentration screenings using inertial microfluidics for studying tumor cell–drug interactions will be studied in our future experiments. We envisage that the device can reduce the complexity of the procedure by establishing stable and effective dual drug-concentration gradients in a flow-rate insensitive microfluidic system for studying tumor cell–drug interactions, thus laying the foundation for the establishment of a low-cost lab-on-a-chip platform in concentration gradient-related biochemical research. 

## Supplementary Materials

The following are available online at https://www.mdpi.com/2072-666X/11/5/493/s1. Figure S1: Comparison of viability in microfluidic methods with conventional methods.
