# Supplementary material for: Concentration Gradient Constructions Using Inertial Microfluidics for Studying Tumor Cell–Drug Interactions
Source: Micromachines (Basel). 2020 May 12;11(5):493. doi: 10.3390/mi11050493 (PMC7281261; doi:10.3390/mi11050493)
Supplement: Supplementary file 1 [file micromachines-11-00493-s001.pdf]

micromachines

MDPI

Supplementary Materials

Concentration Gradient Constructions Using Inertial Microfluidics for Studying Tumor Cell–Drug Interactions

Shaofei Shen, Fangjuan Zhang, Mengqi Gao and Yanbing Niu

1. Device Fabrication

The device used in this research was obtained by soft lithography on a silicon substrate with a standard AZ50XT master mold. First, a microfluidic device containing a spiral mixer and microchamber was designed using AutoCAD software. Next, the above elements were printed on a glass substrate to form a chromium mask (MicroCAD Photomask Ltd., Suzhou, China). Finally, AZ50XT photoresist was used on the BG401A mask calibrator (7 mW·cm^{-2}, CETC, Beijing, China) to prepare the 50 μm high mold under ultraviolet (UV) light. Before manufacturing the microfluidic device, the mold was exposed to trimethylchlorosilane vapor for 3 min. A well-mixed poly (dimethylsiloxane) (PDMS) prepolymer (RTV 615 A and B (10:1, w/w)) was poured into a petri dish to produce a 3 mm thick PDMS replica. After degassing, the mold was cured on a hot plate at 80 °C for 50 min. The PDMS replica was then peeled from the mold. The inlet and outlet holes are punched with metal pins. Afterward, the PDMS replica was trimmed, cleaned, and placed on a clean glass slide coated (3000 rpm, 60 s, ramp 15 s) with a PDMS pre-polymer (RTV 615 A and B (15:1, w/w)) that had been cured for 20 min in an oven at 80 °C. Finally, the microfluidic device was ready for use after baking at 80 °C for 48 h.

2. Numerical Simulation

To evaluate fluid motion in the microfluidic system, computational fluid dynamics (CFD) simulation was performed using ESI-CFD software (V2010.0, ESI CFD, Inc., Huntsville, AL, USA). Different flow rates were specified at the input, and the outlet was set to a fixed pressure boundary condition. No slip boundary condition was applied at the channel walls. FLOW and CHEM modules in CFD-ACE+ were used to explore fluid phenomena in the microchannels. Based on the finite volume method, the conservation of Navier–Stokes momentum in the device is described by Equation S(1) as follows:

$$\frac{\partial}{\partial t}\left(\rho\vec{V}\right) + \nabla\cdot\left(\rho\vec{V}\vec{V}\right) = -\nabla P + \bar{\bar{\tau}} \quad (1)$$

The conservation of mass is described by the continuity equation, Equation S(2), as follows:

$$\frac{\partial\rho}{\partial t} + \nabla\cdot\left(\rho\vec{V}\right) = 0 \quad (2)$$

where ρ is fluid density, $\vec{V}$ is velocity vector, P is pressure, $\bar{\bar{\tau}}$ is stress tensor, t is time and $\nabla$is the standard spatial grad operator. The physical properties of water were applied to the fluids participating in the simulation (density ρ = 1000 kg·m^{-3} and dynamic viscosity μ = 10^{-3} kg·m^{-1}·s^{-1}). A diffusion coefficient D = 10^{-10} m^2·s^{-1} was used for the fluids in the simulations. In addition, for fluid mixing calculation, water A and B are set as 0 and 1, respectively. A second-order limiting scheme was used for solving the species diffusion. The convergence limit for mass fraction was set to 10^{-6} and the simulations were run for ≈2000 time steps until flow reached the outlet.

3. Cell Culture and Treatment

Two types of human cancer cell lines, namely human breast adenocarcinoma cells (MCF-7) cells and human cervical carcinoma cells (HepG2) cells, were used to investigate and analyze the feasibility. MCF-7 cells and HepG2 cells were routinely cultured in Dulbecco's modified Eagle's medium (DMEM) supplemented with 10% fetal bovine serum (FBS), 100 U mL^{-1} penicillin and 100 μg mL^{-1} streptomycin. These cells were then grown and maintained in a humidified atmosphere with 5% CO_2 at 37 °C and were normally passaged at a ratio of 1:3 every three days to maintain their exponential growth phase. They were harvested through trypsinization with 0.25% trypsin (Invitrogen) in Ca^{2+} and Mg^{2+} free Hank's balanced salt solution at 37 °C before use. Trypsinization was stopped upon the addition of fresh supplemented DMEM and the cell suspension was centrifuged at 1000 rpm for 2 min, and then diluted to the required concentrations with DMEM.

Before using microfluidic devices for cell culture the equipment needs to be sterilized and flushed. First, the device was rinsed with 75% alcohol and then rinsed with ultrapure water, phosphate-buffered saline (PBS, 0.01 M, pH 7.4), and fresh DMEM. Finally, the culture chamber was treated with 50 mL^{-1} fibronectin (FN) solution and treated at 37 °C for 4 h. The excess FN solution was washed with PBS buffer.

4. Cell Staining and Cell Viability Assay

Cell viability was assessed using an acridine orange/propidium iodide (AO/PI) staining regimen. First, wash with PBS. Then the AO/PI staining solution (10 mg mL^{-1} each in PBS) was introduced into the microchamber for 10 min. Then PBS was added for rinsing. Fluorescence images were collected in each microchamber to assess the viability of cells under different doses of paclitaxel. The collected images were compared with the control images, and the survival rate was calculated using the total number of living cells in the control cavity and the total number of living cells remaining in other cavities after the gradient treatment. The count and measurement objects of image-pro1 Plus 6.0 (Media Cyternetics, Silver Spring, MD, USA) were used to analyze the image to obtain the cell number. Imaging was performed every day to obtain phase contrast, phosphorescence and fluorescence images.

5. Comparison of Viability in Microfluidic Methods with Conventional Methods

Paclitaxel, at increasing concentrations (0 mg·mL^{-1}, 0.125 mg·mL^{-1}, 0.25 mg·mL^{-1}, 0.375 mg·mL^{-1}, 0.5 mg·mL^{-1}, 0.625 mg·mL^{-1}, 0.75 mg·mL^{-1}, 0.875 mg·mL^{-1}, 1 mg·mL^{-1}) were used to compare the activity obtained by our microfluidic method with the conventional method in the 48-well measuring plate. The experiment was carried out to verify the concentration values of each chamber predicted by the simulation. MCF-7 and HepG2 cell suspensions (1.0×10^7 cells/mL) in DMEM were added to the 48-well assay plates. After 12 h, Adherent cells were treated with media containing different concentrations of paclitaxel. After 24 h of incubation, AO/PI fluorescence images were taken.

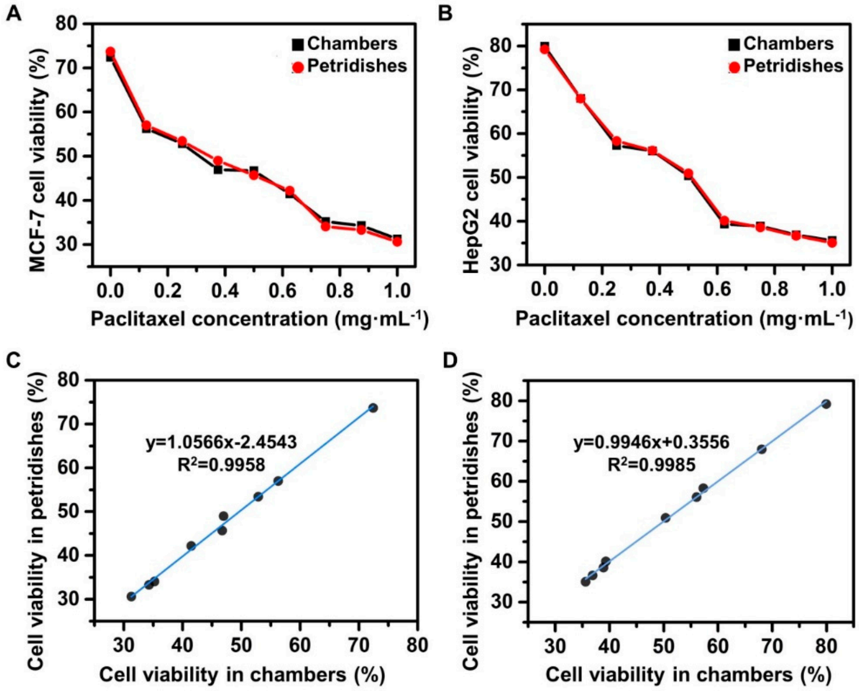

Figure S1. Comparison of viability in microfluidic methods with conventional methods.(**A**,**C**) Comparison of human breast adenocarcinoma cells (MCF-7) cells viability in the microfluidic chip and 9 Petri dishes under different concentrations of paclitaxel treatment (0 mg·mL^{-1}, 0.125 mg·mL^{-1}, 0.25 mg·mL^{-1}, 0.375 mg·mL^{-1}, 0.5 mg·mL^{-1}, 0.625 mg·mL^{-1}, 0.75 mg·mL^{-1}, 0.875 mg·mL^{-1}, 1 mg·mL^{-1}).(**B**,**D**) Comparison of human cervical carcinoma cells (HepG2) viability in the microfluidic chip and 9 Petri dishes under different concentrations of paclitaxel treatment (0 mg·mL^{-1}, 0.125 mg·mL^{-1}, 0.25 mg·mL^{-1}, 0.375 mg·mL^{-1}, 0.5 mg·mL^{-1}, 0.625 mg·mL^{-1}, 0.75 mg·mL^{-1}, 0.875 mg·mL^{-1}, 1 mg·mL^{-1}).

© 2020 by the authors. Licensee MDPI, Basel, Switzerland. This article is an open access article distributed under the terms and conditions of the Creative Commons Attribution (CC BY) license (http://creativecommons.org/licenses/by/4.0/).
